# The role of luteinizing hormone activity in spermatogenesis: from physiology to clinical practice

**DOI:** 10.1186/s12958-024-01333-4

**Published:** 2025-01-13

**Authors:** Sandro C. Esteves, Peter Humaidan

**Affiliations:** 1 ANDROFERT, Andrology and Human Reproduction Clinic, Av. Dr. Heitor Penteado, 1464, Campinas, 13075-460 Brazil; 2 Department of Surgery (Division of Urology), University of Campinas (UNICAMP), Campinas, Brazil; 3 Department of Clinical Medicine, Faculty of Health, Aarhus University, Aarhus, Denmark; 4 Skive Regional Hospital, Fertility Unit, Skive, Denmark

**Keywords:** Male infertility, Hypogonadism, Hormonal therapy, Luteinizing hormone, Human chorionic gonadotropin, Spermatogenesis, Oligozoospermia, Non-obstructive azoospermia

## Abstract

The production of spermatozoa, a process known as spermatogenesis, is primarily controlled by follicle-stimulating hormone (FSH) and luteinizing hormone (LH)-driven testosterone. LH acts on the Leydig cells, stimulating steroid production, predominantly testosterone, and activating critical inter-related spermatogenesis regulatory pathways. Despite evidence that exogenous gonadotropins containing LH activity can effectively restore spermatogenesis in males with hypogonadotropic hypogonadism, the use of these drugs to treat other forms of male infertility is the subject of an ongoing debate. In this review, we delve into the molecular properties and functions of LH activity in spermatogenesis regulation and explore available preparations for therapeutic use. We also examine the evidence regarding the effectiveness of LH-containing drugs in treating specific male infertility conditions and identify the main areas for future research. Our review highlights the critical role of LH in spermatogenesis and emphasizes the potential of LH-containing drugs in treating male infertility. However, further research is required to completely elucidate the mechanisms underlying the effects of LH activity on sperm production and to establish the most effective dosages and treatment durations.

**Supplementary Information:**

The online version contains supplementary material available at 10.1186/s12958-024-01333-4.

## Introduction

Spermatogenesis involves the proliferation and differentiation of spermatogonia into spermatozoa, which is essential for male fertility [1]. The process occurs within the seminiferous tubules of the testicles in close contact with the Sertoli cells. The hypothalamic-pituitary–gonadal axis has a vital function in regulating spermatogenesis through the secretion of gonadotropin-releasing hormone (GnRH), follicle-stimulating hormone (FSH), and luteinizing hormone (LH) [1, 2]. Briefly, neurons in the hypothalamus's periventricular infundibular region secrete GnRH, which travels to the pituitary gland via the hypothalamic-pituitary portal blood system. GnRH triggers the release of LH and FSH into the bloodstream, and both hormones are produced by gonadotropes situated in the anterior pituitary gland.

Normal spermatogenesis relies on both FSH and LH [1]. LH plays a crucial role as it promotes the synthesis of testosterone in Leydig cells, which then binds to androgen receptors on Sertoli cells, modulating gene transcription and secretion of paracrine stimuli for spermatogenesis. LH-driven testosterone acts synergistically with FSH; the latter provides metabolic and structural support for spermatogenesis via its receptors in Sertoli cells [3].

That both FSH and LH are essential for normal spermatogenesis is particularly evident in males with hypogonadotropic hypogonadism (HH) [2]. Clinical studies have shown that in such patients, pharmacotherapy with regimens containing LH activity can effectively restore spermatogenesis in most cases [2, 4]. In HH males, the best results in terms of sperm production are achieved with the co-administration of human chorionic gonadotropin (hCG) and FSH [2, 4].

Although it is widely recognized that exogenous LH activity is crucial for restoring spermatogenesis in hypogonadal men with inadequate LH production or action (e.g., HH males), the role of LH in hypogonadal infertile males with no evident LH deficiency, like patients diagnosed with idiopathic oligozoospermia or non-obstructive azoospermia, is still a subject of controversy [1] and currently an active area of research.

In this narrative review, we aim to (i) provide an overview of the function of LH activity in spermatogenesis regulation, (ii) summarize the evidence for the therapeutic use of preparations containing LH activity in males with infertility, and (iii) outline the main areas for future research. By exploring the latest research in this area, we hope to clarify the potential benefits and limitations of LH-activity usage for male infertility treatment and pave the way for further advancement in this field.

### Luteinizing hormone: structure and role in spermatogenesis

#### The molecule

LH is a glycoprotein containing two non-covalently linked subunits, α and β. The α subunit, which is identical for LH, FSH, and hCG, consists of 92 amino acids [5]. In contrast, the β subunits of LH, FSH, and hCG are distinct, providing receptor specificity and different biological properties. The LHβ subunit, which is made up of 121 amino acids, is produced from mRNA transcripts encoded by the *LHB* gene located on chromosome 19q13.32. The biological activity and half-life of the LHβ subunit are influenced by the addition of carbohydrate molecules, leading to the formation of heterodimers [5]. The LHβ subunit contains a sole N-linked glycosylation site at asparagine 30 and one or two sialic acid residues. The terminal half-life of endogenous LH is brief (20–30 min).

The *LHβ* gene is located in a genetic cluster that also encodes for the β subunit of hCG i [1]. The *LHβ* and *hCGβ* genes share around 95% similarity, with the main discrepancy being an additional sequence of chorionic gonadotropin beta in *hCGβ*. Consequently, the hCG molecule has a 28-amino acid extension with five additional glycosylation sites [5].

#### Physiology

LH attaches to transmembrane receptors (LHCGR) situated on Leydig cells, initiating the process of testosterone synthesis. Specifically, LH promotes the transcription of genes that encode enzymes implicated in steroidogenic pathways. Moreover, LH-mediated downstream activities trigger the production of growth factors by Leydig cells, which are essential for spermatogonia proliferation [1].

Testosterone production, which is primarily regulated by LH, is crucial for spermatogenesis. Intratesticular testosterone (ITT) binds intracellular androgen receptors located on Sertoli cells, stimulating the secretion of paracrine stimuli necessary for germ cell development [6]. The primary function of ITT is to promote the development of round spermatids into mature sperm during spermiogenesis. Furthermore, ITT helps transition type A to type B spermatogonia and upregulates androgen receptor expression, thereby improving Sertoli cell function [6]. Testosterone can undergo partial conversion to estradiol via aromatase or dihydrotestosterone via 5α-reductase.

LH-driven testosterone works in synergy with FSH to increase sperm quantity. Specifically, FSH regulates structural genes responsible for cell–cell junction organization and genes implicated in transporting regulatory and nutritive molecules from Sertoli cells to germ cells [3]. FSH also controls the proliferation of Sertoli cells, supports their growth and maturation, and triggers the release of androgen-binding protein. Although not required for spermatogenesis completion in humans, FSH deficiency significantly reduces sperm production [1, 3].

#### Mutations in the *LH* and *LHCGR* genes

Naturally occurring mutations in the *LH* and *LHCGR* genes in humans are uncommon, but they underscore the crucial role of this hormone and its receptor in spermatogenesis. Several case reports have demonstrated that complete inactivating mutations of *LHβ* do not seem to affect LH dimerization or immunoreactivity. However, they lead to a lack of pubertal development, hypogonadism, and infertility due to the absence of functional pituitary LH and subsequent impaired testosterone production [7–10]. Although these patients are masculinized at birth, their semen analyses reveal azoospermia, and testicular biopsies show absent (or very few) Leydig cells and arrested germ cell maturation.

An interesting case report described a deletion of the exon 10-coded portion of the *LHCGR* gene [11]. The patient exhibited Leydig cell hypoplasia, delayed pubertal development, small testes, high serum LH, and low testosterone levels. He did not respond to endogenous LH, but the administration of exogenous hCG restored androgen biosynthesis and spermatogenesis. In vitro analysis confirmed the absence of LH-induced cAMP increase, while hCG activated the signal transduction pathway upon binding to the mutant receptor [12].

These findings indicate that in humans, LH is not essential for the differentiation of the male external genitalia. Instead, hCG, produced by the placenta and homologous to LH, allows the masculinization of the genitalia before birth by acting on LHCGR [3, 13]. However, after birth, LH plays a crucial role in testicular maturation by promoting the proliferation and maturation of Leydig cells These cells secrete testosterone, which is essential for pubertal virilization and the initiation of spermatogenesis [13]. While hCG therapy has shown to successfully restore spermatogenesis to normal levels in patients with *LHβ* mutations [4, 12, 14], a case involving a patient in whom high doses of hCG were unable to restore spermatogenesis suggests the existence of an essential and timely ‘window of testicular susceptibility’ for the proliferation and differentiation of Leydig cells [15].

#### Drugs containing LH activity

Drugs containing LH activity have been available for a long time. HCG and human menopausal gonadotropin (hMG) were first extracted from urine in the 1940s, and by the 1950s, the first urinary forms of hCG and hMG became commercially available. In 2001, advances in DNA technology enabled the development of recombinant (r) human LH and hCG, introducing new possibilities for drug administration. These advancements also facilitated the development of the filled-by-mass method, which ensures precise measurement of protein levels in recombinant gonadotropin products, and as well as the creation of pen devices designed for their administration medications [5]. The characteristics of commercially available drugs gonadotropins containing LH are shown in Table 1.

**Table 1:** Characteristics of commercially available gonadotropins containing LH

	**Purity** **(LH content)**	**FSH activity** **(IU/vial)**	**LH activity** **(IU/vial)**	**hCG content** **(IU/vial)**	**Specific activity** **(LH/mg protein)**
Lutropin alfa	> 99%	0	75^a^	–	9000
Follitropin alfa + lutropin alfa 2:1 ratio	> 99%	150	75	–	9000
HP-hMG	Unknown^b^	75	75^b^	~ 8	–

*FSH* follicle-stimulating hormone, *hCG* human chorionic gonadotropin, *HP-hMG* highly purified human menopausal gonadotropin, *LH* luteinizing hormone

^a^1 µg of lutropin alfa = 22 IU

^b^Derives primarily from the hCG component, which is concentrated during the purification process or sometimes added to achieve the desired amount of LH-like biological activity

Urinary hCG preparations are commonly available in lyophilized vials for intramuscular or subcutaneous use, with doses ranging from 1000 to 10,000 IU [5]. On the other hand, rhCG is supplied in ready-to-use syringes and pen devices containing 250 µg of pure hCG. This amount equals ~6500 IU of urinary hCG (Supplementary Table 1). While recombinant hCG formulations have long been used clinically for ovarian stimulation, their use in male infertility treatment has only recently been reported [1, 16, 17].

#### Differential molecular action of LH and hCG in Leydig cells

**Fig. 1:**
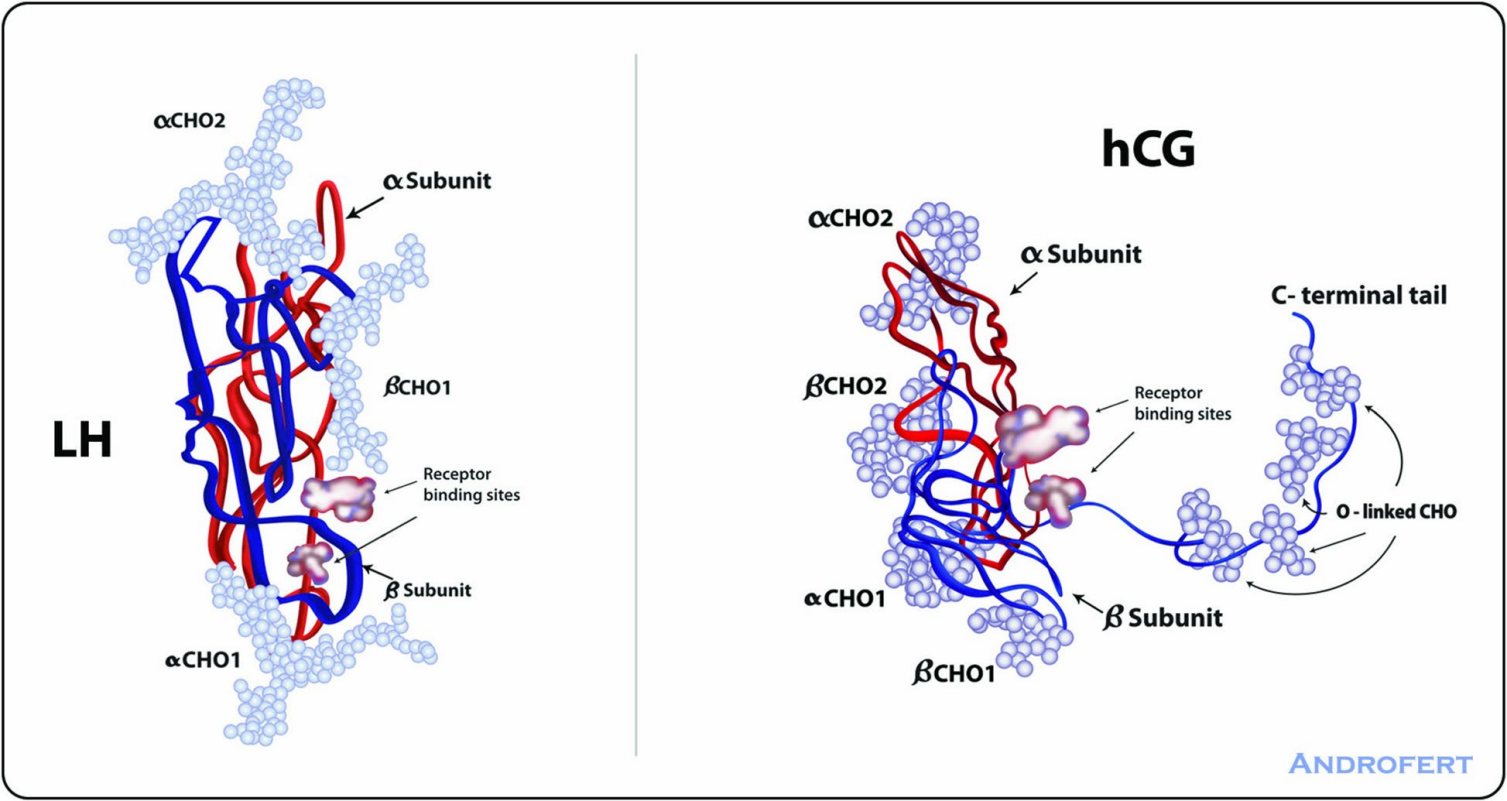
Luteinizing hormone (LH) and human chorionic gonadotropin (hCG) have similar structural features. LH (left panel) is a glycoprotein consisting of two subunits, the α subunit and the β subunit (blue). The α subunit is similar to that of follicle-stimulating hormone (FSH) and hCG, with two carbohydrate attachment sites. On the other hand, the β subunit has only one carbohydrate attachment site. The Though structurally similar to LH, hCG (right panel) has a notable difference: it contains a long carboxy-terminal segment that is O-glycosylated (O-linked CHO), which gives hCG a longer half-life. In the illustration, the α and β subunits are represented by red and blue strands, respectively, with light blue balls representing the carbohydrate chains. Adapted from Leão Rde B, Esteves SC. Gonadotropin therapy in assisted reproduction: an evolutionary perspective from biologics to biotech. Clinics (Sao Paulo). 2014;69(4):279–93. This article is distributed under the Creative Commons Attribution License (CC BY 4.0)

**Fig. 2:**
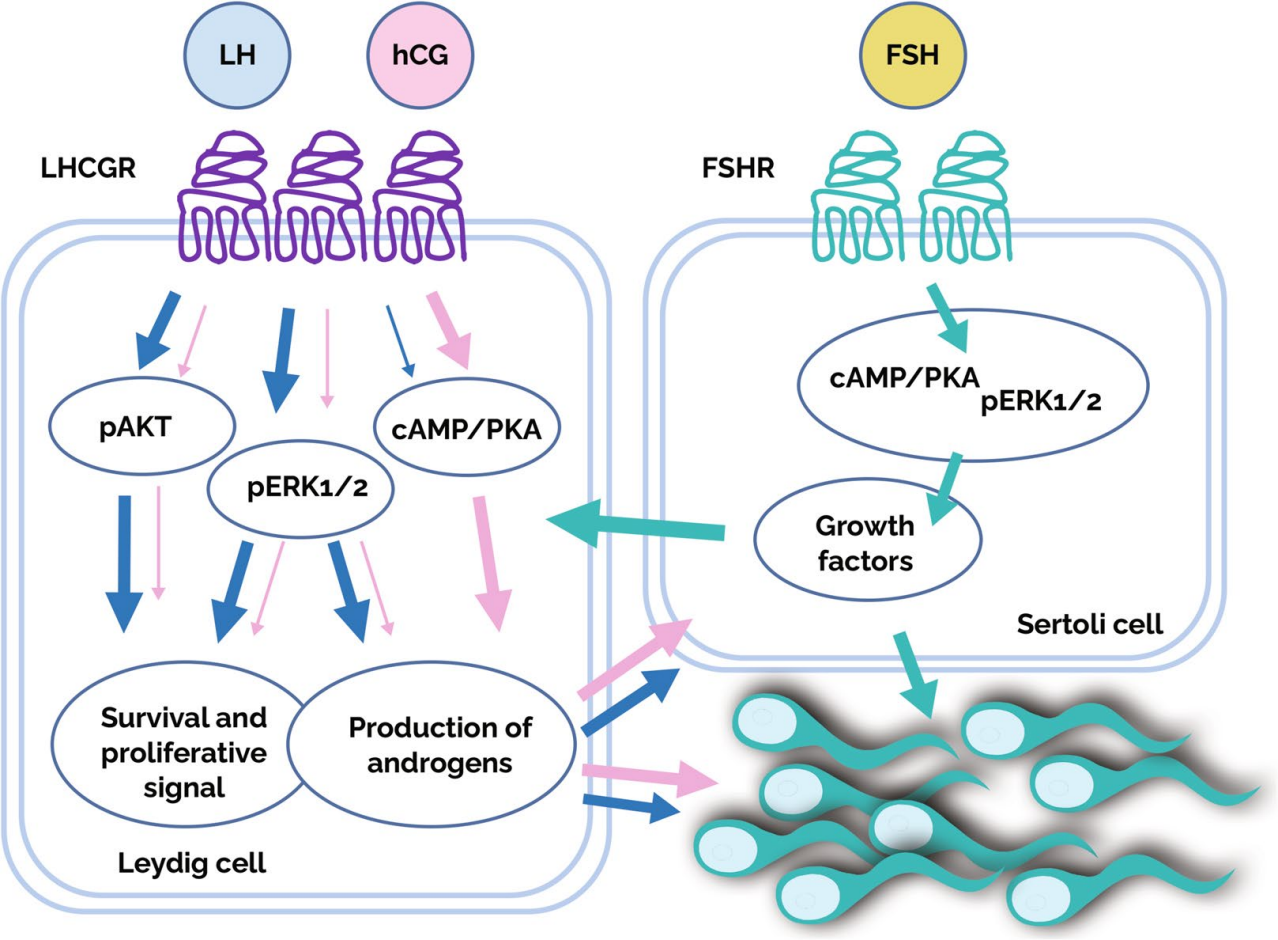
Gonadotropins such as LH and hCG act on the male gonads by targeting the Leydig cell-specific receptor, quantitatively and qualitatively activating different intracellular signaling pathways. LH activates survival and proliferative events, whereas hCG triggers mainly steroidogenic signals. LH signals are activated by the recruitment of G protein and b-arrestins, leading to the phosphorylation of ERK1/2 and AKT and resulting in proliferative and antiapoptotic events. On the other hand, hCG is more potent than LH for inducing the activation of both cAMP/PKA and pERK1/2 pathways. Nevertheless, both molecules have a similar balance of stimulatory and inhibitory steroidogenic signals that enhance testosterone synthesis required for spermatogenesis. The width of the arrows indicates the differential activity of LH (blue arrows) and hCG (pink arrows) (see text). Reprinted with permission from Elsevier from Esteves et al., Male infertility and gonadotropin treatment: what can we learn from real-world data? Best Pract Res Clin Obstet Gynaecol. 2023;86:102,310

The β subunit structural differences between LH and hCG result in distinct molecular actions in the Leydig cell. HCG has a longer carboxy-terminal segment and more glycosylation sites than LH, impacting receptor affinity and half-life [5] (Fig. 1). HCG and LH bind to the same LHCGR receptor on the Leydig cells, but the former does so with greater affinity and has a longer half-life. The terminal half-life of hCG, administered intravenously, is 24 h, whereas the correspondent half-life of LH is ~ 30 min. When rhLH is administered subcutaneously, the drug has a shorter terminal half-life (10–12 h) than the 24–31 h needed for hMG preparations with hCG-driven LH activity to be eliminated [5]. The LHCGRs may discriminate between the two ligands as hCG preferentially induces cAMP and increases intracellular Ca2 + , leading to the upregulation of genes that code steroidogenic enzymes [1]. On the other hand, LH has lower steroidogenic potency than hCG and it activates mitogenic signals through ERK1/2 or AKT phosphorylation, which can improve cellular metabolic state [1] (Fig. 2).

Limited data exist on the role of rhLH and hCG administered exogenously on Leydig cell function, particularly in human male gametogenesis. However, a few clinical studies have suggested that LH and hCG have distinct actions on Leydig cells and that even low rhLH doses may be effective in inducing androgen production in vivo [11, 18–20], as summarized in Table 2.

**Table 2:** Studies exploring the action of luteinizing hormone and/or human chorionic gonadotropin in human Leydig cells

Study	Study design and population	Methods	Main Results
Gromoll et al. (2000) [11]	Case report of an 18-year-old boy with a deletion of the exon 10-coded portion of the *LHCGR* gene (autosomal recessive inheritance)	The patient had retarded pubertal development, small testicles, and delayed bone maturation. LH was highly elevated (29.2 IU/L), with very low serum T levels (3.9 nmol/L). LH bioactivity was tested using a mouse Leydig cell bioassay and was normal, indicating a complete resistance to LH action. An hCG stimulation test (5000 IU/m^2^) was carried out, and hCG treatment was continued for 16 wk (5000 IU/wk for 2 mo, then reduced to 3000 IU/wk)	The patient was unresponsive to endogenous LH, but exogenous hCG administration restored androgen biosynthesis to the normal range. Prolonged hCG treatment resulted in an increase in testicular volume and the appearance of spermatozoa in the ejaculate after 16 wks of treatment (5.3 million/mL). In vitro analysis confirmed the lack of LH-induced cAMP increase, while hCG activated the signal transduction pathway on mutant receptor binding
Cailleux-Bounacer et al. (2008) [18]	Randomized, single-blind study including 20 healthy eugonadal men aged 18–30 y	A subgroup received either vehicle alone or urinary hCG IM in doses varying from 50 to 5000 IU, while another group received recombinant hCG IM in a dose of 250 µg (~6500 IU) or recombinant LH IV in doses varying from 75 to 225 IU. A serial assessment of serum T and E2 was carried out to determine the response to stimulation	There was a dose-dependent increase in T and E2 levels with urinary hCG administration, with a peak observed 48 and 72 h after the injection of 500 and 5000 IU, respectively. The peaks in T and E2 obtained with urinary hCG (5000 IU) and rhCG (6500 IU) did not differ Serum LH levels increased dose-dependently after injection of recombinant LH; the peak value was obtained 30 min after injections, progressively dropping to reach basal values 6 h later. Similarly, a dose-dependent increase in T (but not E2) was observed after rLH administration, with a peak observed 6 h after administering 225 IU The highest serum T levels attained within 5 h after injecting either 150 or 225 IU rLH correlated with the 48-h peak increase in T in response to the injection of 50 IU rhCG. These findings confirm that recombinant hCG and recombinant LH promote adequate androgen production in normal men
Santi et al. (2017) [19]	Case report of a 55-year-old man with an atypical giant FSH-secreting pituitary adenoma who underwent hypophysectomy and became hypogonadotropic, hypogonadal, and azoospermic	Three schemes were consecutively administered: (i) highly purified urinary hCG 75 IU daily IM for 2 wks; (ii) recombinant LH (rhLH) 75 IU daily IM for 2 wks; (iii) hCG 75 IU daily IM for 2 wks. Each stimulation scheme was followed by a 2-wk washout period. The initial hCG administration was performed to sensitize the Leydig cells to subsequent stimulations. The subsequent rhLH and hCG administrations were conducted to compare the in vivo response to the two gonadotropins. Serum total T levels were measured by isotopic dilution/LC–MS	All three regimens led to a significant increase in serum total T levels vs baseline. The maximal T increase was not significantly different between hCG (3.46 ng/mL) and rhLH (2.49 ng/mL; *p* = 0.245). T increased immediately after the first injection, and it maintained a steady state by both approaches (rhLH and hCG). LH serum levels increased after rhLH administration from 2.7 to 5.7 mIU/mL, suggesting that a minimal, apulsatile LH increase was able to stimulate Leydig cells
Veldhuis et al. (2012) [20]	Controlled trial involving 19 healthy men	Patients received a selective GnRH antagonist (Ganirelix) overnight for pituitary suppression, followed by saline or rhLH IV continuously or as 6-min pulses IV every 1 or 2 h at the same total dose (112.5 IU). Blood was sampled every 10 min for 10 h to quantify T responses using LC–MS	Relatively low doses of recombinant LH could was able to exert Leydig cell stimulation. A pulsatile LH signal, though not required for mean T output, strongly determined T secretion patterns. Accordingly, 1- and 2-h rhLH pulses evoked higher mean LH and T concentrations than a continuous infusion of the same amount of rhLH

*CG* chorionic gonadotropin, *E2* estradiol, *FSH* follicle-stimulating hormone, *GnRH* gonadotropin-releasing hormone, *h* human, *IM* intramuscular, *IV* intravenous, *LC–MS* liquid chromatography–mass spectrometry, *LH* luteinizing hormone, *mo* month[s], *r* recombinant, *T* testosterone, *wk* week[s]

### Therapeutic use of gonadotropins containing LH activity in male infertility

Historically, hCG has been the primary gonadotropin utilized in infertile males with hypogonadism [1, 2, 16, 17, 21]. The administration of hCG as a surrogate for LH to remediate male infertility conditions associated with hypogonadism, like HH and non-obstructive azoospermia (NOA), is founded on the notion that reduced ITT levels can disrupt spermatogenesis [6]. In rodents, ITT concentrations must remain above 25% of normal levels, as reductions exceeding 75% are incompatible with sperm maturation [22]. It is important to note that the action of endogenous LH and FSH depends on the frequency, amplitude, and duration of their secretory pulses. In HH males, the levels of both gonadotropins are markedly reduced. By contrast, NOA males with spermatogenic failure typically have elevated circulating levels of FSH and LH. As a result, their secretion profile is characterized by relatively low amplitudes, leading to weak stimulation of Sertoli and Leydig cells [2, 23]. Studies conducted in men with NOA have demonstrated that hormonal therapy utilizing hCG can elevate ITT levels and promote the synthesis of spermatogonial DNA [23]. Additionally, the high circulating FSH levels may cause pathological desensitization of the FSH receptor [24]. For such patients, hormonal therapy using GnRH or hCG has been suggested as a potential solution to suppress the elevated endogenous gonadotropin levels and counteract the Sertoli cell receptor desensitization triggered by the chronically high circulating levels of endogenous FSH [25].

#### Hypogonadotropic hypogonadism

HH is a medical condition resulting from congenital or acquired disorders that affect the hypothalamus and/or the pituitary gland [2]. The congenital form of HH comprises anosmic HH (Kallmann syndrome) and normosmic isolated HH (idiopathic hypogonadotropic hypogonadism). Acquired HH can result from various causes, including the use of certain drugs (e.g., anabolic steroids and testosterone replacement therapy), infectious or infiltrative pituitary lesions, hyperprolactinemia, encephalic trauma, pituitary/brain radiation, excessive exercise, substance abuse involving alcohol or illicit drugs, and systemic diseases such as hemochromatosis, sarcoidosis, and histiocytosis X [2].

Regardless of its cause, HH is characterized by inadequate stimulation of the testes due to insufficient pituitary gonadotropin secretion. In adults, the clinical features of HH include androgen deficiency and infertility [2]. Levels of circulating FSH, LH, and T are typically low (e.g., FSH < 1.5 IU/L, LH < 1.5 IU/L, T < 300 ng/dL), and the semen analysis shows azoospermia or severe oligozoospermia.

For males with HH who wish to obtain biological fatherhood, therapeutic options include GnRH pumps or the administration of gonadotropins containing LH activity. Gonadotropin therapy with hCG alone or in combination with hMG, urinary FSH, or rFSH, has been shown to restore spermatogenesis (to varying degrees) in up to 90% of patients. Moreover, the reported pregnancy rates with this treatment have reached as high as 65% with natural or assisted methods [2, 4].

Therapeutic regimens vary but typically begin with administering 1000–2500 IU of hCG twice weekly for 8–12 weeks. This initial phase is critical for increasing ITT levels. HCG alone can reinstate complete spermatogenesis, especially in adult-onset HH. However, for patients with congenital HH or adult-onset HH that is refractory to hCG monotherapy, treatment the simultaneous administration of FSH, 150–225 IU, two to three times weekly for up to 18 months [2, 4]. Sperm concentration is typically higher when hCG is combined with FSH compared to hCG alone however, there is a lack of trials directly comparing drugs and regimens. During treatment, patients should be monitored through hormone testing and semen analysis, and sperm banking is recommended for those who respond to therapy [26].

#### Idiopathic oligozoospermia

Men with idiopathic oligozoospermia, defined by reduced sperm concentration without a clear underlying cause, and unremarkable findings on physical examination and endocrine laboratory results, pose a challenge for clinicians. Various studies have examined the efficacy of hormonal treatment for this type of patient, with particular emphasis on FSH monotherapy (reviewed in [1]). However, a limited number of trials have investigated the use of LH-activity gonadotropins [27–31], as summarized in Table 3. While most studies report improvements in semen parameters and increased pregnancy rates, these findings should be interpreted with caution due to small sample sizes, variabilithy in gonadotropin therapy regimens, treatment durations, and follow-up periods. Furthermore, most trials did not account for the prevalence of hypogonadal men in the treated population and relied solely on pre- and post-semen analysis, often with short follow-up periods. Further research is needed to clarify the benefits of gonadotropin therapy in this specific patient population.

**Table 3:** Characteristics of studies assessing the clinical utility of LH-activity containing gonadotropins for males with idiopathic oligozoospermia

Study	Study design	Country	Study population	Mean age (SD or range)	Intervention regimen	Mean Tx period	Control group	Semen parameters outcomes	Pregnancy outcomes	Adverse events
Andrabi et al. (2022) [27]	CS	India	*N* = 56 Hypogonadal patients (TT < 400 ng/dL) with idiopathic severe O (< 5 million/mL)	▪ Responder group: 32.0 (4.3) Non-responder group: 30.4 (3.3)	rhCG 1 × /wk	6 mo	No	Improvement in sperm concentration and TSC; motility unchanged	NR	NR
Schill et al. (1982) [28]	RC	Germany	*N* = 48 NG (FSH levels < 3.5 ng/mL; TT > 3.0 ng/mL) with idiopathic O (< 20 million/mL) ▪ Group A (*n* = 9): sperm concentration 1–5 million/mL ▪ Group B (*n* = 12): 5.1–10 million/mL ▪ Group C (*n* = 18): 10.1–15 million/mL Group D (*n* = 9): 15.1–20 million/mL	34.4 ▪ (5.0)	hMG 75 IU 3 × /wk + uhCG 2500 IU 2 × /wk	3 mo	No	Increased sperm concentration, TSC and progressive motility; morphology unchanged	Data of 33 patients available; natural pregnancies ≤ 1 y after initiation of treatment ▪ Overall PR: 10/33 (30.3%) ▪ Group A: 4/7 (57.1%) ▪ Group B: 1/7 (14.3%) ▪ Group C: 5/12 (41.7%) ▪ Group D: 0/7 (0%)	NR
Knuth et al. (1987) [29]	RCT	Germany	*N* = 37 NG (hormonal profile not specified) with idiopathic O (< 10 million/mL) ▪ IG: *n* = 17 ▪ CG: *n* = 20	▪ IG: 31.1 (3.6) ▪ CG: 33.2 (6.5)	hMG 150 IU 3 × /wk + uhCG 2500 IU 2 × /wk	13 wk	Yes	No difference in sperm concentration and motility between groups	▪ IG: 2/17 (11.7%) ▪ CG: 0/20 (0%)	Breast tenderness and gynecomastia (1 case)
Foresta et al. (2009) [30]	RCT	Italy	*N* = 87 HG (FSH threshold not specified) with idiopathic and non-idiopathic severe O (< 3 million/mL) and testicular histopathology showing HYPO ▪ IG: *n* = 57 ▪ CG: *n* = 30	34.2 (4.5)	Leuprolide acetate 3.75 mg 1 dose, and after 30 d, leuprolide acetate 3.75 mg 1 × /mo + rFSH 150 IU every 2 d + hCG 2000 IU (type not specified) 2 × /wk	3 mo	Yes	Increased sperm concentration and morphology in treatment group vs control group, but motility unchanged	Natural pregnancy: ▪ IG: 4/57 (7%) ▪ CG: 0/30 (0%) ART: ▪ IG: IVF 6/21 (28.6%) ▪ ICSI: 8/32 (25.0%) ▪ CG: only ICSI 6/30 (20.0%) Total PR: ▪ IG: 18/57 (31.6%) ▪ CG: 6/30 (20.0%)	10 patients experienced AEs related to androgen deprivation (asthenia, hot flushes, headache) that disappeared after hCG administration
La Vignera et al. (2020) [31]	CS	Italy	*N* = 210 NG or hypogonadal (TT < 350 ng/dL) with OA (< 15illion/mL)	30.7	Various regimens including: ▪ Group A (*n* = 40; TV > 12 mL + TT > 350 ng/dL): FSH 150 IU (type not specified) 3 × /wk for ≥ 3 mo ▪ (ii) Group B (*n* = 60; TV > 12 mL + TT < 350 ng/dL): hCG 2000 IU (type not specified) 2 × /wk for 3 mo; if no sperm parameters improvement, then FSH added for 3 mo; if TT remained low, then hCG continued for another 3 mo; if TT normalized, hCG suspended ▪ Group C (*n* = 65; TV < 12 mL + TT < 50 ng/dL): FSH 150 IU (type not specified) 3 × /wk + hCG 2000 IU (type not specified) 2 × /wk ▪ Group D (*n* = 45; TV < 12 mL + TT > 350 ng/dL): FSH 150 IU (type not specified) 3 × /wk for 3 mo, if TV increased, but no improvement in sperm parameters, hCG 2000 IU added (type not specified) 1 × /wk for 3 mo; if TV and sperm parameters unchanged, FSH continued for another 3 mo	3–6 mo	No	Improvement in parameters and decreased sperm DNA fragmentation rates	Natural pregnancy: ▪ Group A: 15/60 (25%) ▪ Group B: 12/40 (30%) ▪ Group C: 20/65 (31%) ▪ Group D: 15/45 (33%)	NR

*AE* adverse event, *ART* assisted reproductive technology, *CG* control group, *CS* case series, *FSH* follicle-stimulating hormone, *Hcg* human chorionic gonadotropin, *HG* hypergonadotropic, *hMG* human menopausal gonadotropin, *HYPO* hypospermatogenesis, *ICSI* intracytoplasmic sperm injection, *IG* intervention group, *IVF* in vitro fertilization, *mo* month[s], *NG* normogonadotropic, *NR* not reported, *O* oligozoospermia, *OA* oligoasthenozoospermia, *PR* pregnancy rate, *RC* retrospective cohort study, *RCT* randomized controlled trial, *rFSH* recombinant follicle-stimulating hormone, *rhCG* recombinant human chorionic gonadotropin, *TSC* total sperm count, *TT* total testosterone, *TV* testicular volume, *uhCG* urinary human chorionic gonadotropin, *wk* week[s], *y* year[s]

#### Non-obstructive azoospermia

NOA is a medical condition characterized by severe and untreatable intrinsic testicular defects that adversely affect sperm production [17, 21]. This condition can be caused by genetic and congenital abnormalities, post-infectious testicular damage, exposure to gonadotoxins, and testicular trauma. Additionally, NOA can be idiopathic, where the underlying cause cannot be determined. Men diagnosed with NOA typically display spermatogenic failure, evidenced by the absence of sperm in the ejaculate, high serum FSH levels, and small testicles. In most cases, biochemical hypogonadism is also present [17]. However, about 30–60% of NOA patients have focal sperm production in their testes [17, 21, 32]. Retrieval of sperm from the testis is possible, and such sperm can be used for intracytoplasmic sperm injection (ICSI), which is currently the only viable alternative for men with NOA to father their biological progeny [16, 33, 34].

Gonadotropins with LH activity have been used off-label to regulate male reproductive hormones and enhance ITT levels in hypogonadal NOA males [1, 6, 17, 21, 23]. The goals are to increase the success rates of surgical sperm retrieval and the chances of finding sperm in the ejaculate. HCG has been the primary medication used due to its beneficial effect on ITT production and spermatogonial DNA synthesis [23]. Studies assessing the effectiveness of LH activity–containing gonadotropins in men with NOA have shown an overall positive impact of therapy on surgical sperm retrieval rates [1, 6, 17, 21, 32, 35]. However, the available data on the efficacy of the treatment is limited and mainly derived from case series and cohort studies [27, 36–48], as summarized in Table 4. While some studies have reported the successful return of sperm in the ejaculate with the treatment [16], more research is needed to validate its effectiveness.

**Table 4:** Characteristics of studies reporting the use of gonadotropin therapy containing LH activity for males with nonobstructive azoospermia

Study	Study design	Country	NOA population	Mean age (SD or range)	Intervention regimen	Mean Tx period	Control group	SR method	Sperm return to ejaculate, n (%)	Sperm retrieval success, n (%)	Pregnancy data	Adverse events
Laursen et al. (2022) [16]	CS	Denmark	*N* = 8 Mixed population of NG and HG with failed TESA	36.3 (28.0–45.0)	rhCG 1620 IU 2 × /wk for 1 mo, followed by (i) same regimen with (*n* = 1; T/E2 ratio < 10) or without addition of anastrozole 1 mg/d (*n* = 1), or (ii) rFSH added (150–225 IU) 2 × /wk if follow-up FSH levels < 1.5 IU/L; *n* = 6)	10 mo (range: 8–15)	No	TESA	2/8 (25%)	2/8 (25%)	▪ PR: 4/8 (50%) ▪ OPR/LBR: 3/8 (37.5%)	None
Andrade et al. (2021) [17]	CR	Brazil	*N* = 2 NG with MA ▪ Case 1: FSH: 6.1 mIU/mL; TT: 266 ng/dL ▪ Case 2: FSH: 4.4 mIU/mL; TT: 360 ng/dL	▪ Case 1: 36 ▪ Case 2: 35	▪ Case 1: rhCG 125 µg 2 × /wk for 2 months, followed by rFSH 150 IU 2 × /wk + anastrozole 1 mg/d for 4 mo ▪ Case 2: rhCG 125 µg 2 × /wk + rFSH 150 IU 2 × /wk for 5 mo	4–5 mo	No	mTESE	▪ Case 1: no sperm after 6 months of Tx ▪ Case 2: rare abnormal motile sperm after 5 mo of Tx	2/2 (100%)	▪ Case 1: Singleton delivery from ICSI using testicular sperm ▪ Case 2: No pregnancy	NR
Andrabi et al. (2022) [27]	CS	India	*N* = 58 HG hypogonadal (TT < 400 ng/dL)	31.4 (3.7)	rhCG 6500 IU 1 × /wk	≥ 3 mo	No	NA	25/58 (43.1%)	NR	▪ LBR (natural pregnancy): 4/25 (16%)	Weight gain (1 case)
Schiff et al. (2005) [36]	CS	USA	*N* = 42 HGH (mean FSH: 33.2 IU/L; mean TT: 190.2 ng/dL) and HG eugonadal, mostly non-mosaic KS (*n* = 54 SR attempts) ▪ IG: *n* = 36 (HGH patients) CG: *n* = 6 (eugonadal patients)	32.8 (24– 52)	Various regimens: ▪ Testolactone 50–100 mg 2 × /d + uhCG 1500 IU 2 × /wk (hCG dose titrated based on TT levels with maximum dose of 2500 IU 3 × /wk; *n* = 13) ▪ Testolactone 50–100 mg 2 × /d (*n* = 19) ▪ Anastrozole 1 mg/d (*n* = 5) ▪ Anastrozole 1 mg/d + hCG (regimen not specified) (*n* = 1) ▪ CC 25 mg/d (*n* = 3) rFSH (regimen not specified) (*n* = 1)	4 mo	Yes	mTESE	NR	IG: 25/42 (69.4%) ▪ Anastrozole: 5/5 (100%) ▪ Anastrozole + hCG: 1/1 (100%) ▪ CC: 3/3 (100%) rFSH: 1/1 (100%) ▪ Testolactone: 14/19 (74%) ▪ Testolactone + hCG: 7/13 (54%) ▪ Unknown treatment: 2/6 (33%) CG: 4/6 (66.6%) *p* value NR	▪ PR: 18/29 (62%) (21 babies born); pregnancy data NR by type of treatment	NR
Selman et al. (2006) [37]	CS	Italy	*N* = 49 NG (hormonal profile not specified) with MA	32–41	rFSH 75 IU EOD for 2 mo, followed by rFSH 150 IU EOD for 1 mo, and lastly, rFSH 150 IU EOD + uhCG 2000 IU 2 × /wk for 3 mo	6 mo	No	cTESE	0/49 (0%)	11/49 (22.4%)	▪ CPR: 3/11 (27.2%) ▪ LBR: 3/11 (27.2%)	NR
Shiraishi et al. (2012) [38]	RC	Japan	*N* = 48 Mixed population of NG and HGH (mean FSH: 28 IU/L; mean TT: 372 ng/dL) with failed TESE ▪ IG: *n* = 28 ▪ CG: *n* = 20	▪ IG: 34.0 (5.7) ▪ CG: 33.0 (4.9)	uhCG 5000 IU 3 × /wk for 3 mo (*n* = 28) and continued for another 1–2 mo if follow-up FSH levels > 3 IU/L (*N* = 13); or rFSH added (150 IU 3 × /wk for 2 mo) if follow-up FSH levels dropped to < 3 IU/L (*n* = 15)	3–6 mo (range)	Yes	mTESE	NR	▪ IG: 6/28 (21.4%) ▪ CG: 0/20 (0%) *▪ p* < 0.05	NR	Acne: 3/28 (10.7%); Gynecomastia 2/28 (7.1%)
Reifsnyder et al. (2012) [39]	RC	USA	*N* = 388 Mixed population of eugonadal and hypogonadal men (TT < 300 ng/dL; *n* = 348) ▪ IG: *n* = 307 (hypogonadal) ▪ CG: *n* = 41 (hypogonadal) and *N* = 388 (eugonadal)	▪ IG: 34.0 (4.0) ▪ CG: 37.0 (3.0)	Various regimens: ▪ Anastrozole 1 mg/d (*n* = 180) ▪ Anastrozole 1 mg/d + uhCG 1500–2000 IU 2–3 × /wk (*n* = 29) ▪ CC (*n* = 66; regimen not specified) ▪ Testolactone 50–100 mg 2 × /d (*n* = 14) ▪ Testolactone 50–100 mg 2 × /d + uhCG 1500–2000 IU 2–3 × /wk (*n* = 12); ▪ uhCG 1500–2000 IU ▪ 2–3 × /wk (*n* = 9) ▪ Other combinations/ unknown (*n* = 38)	2–3 mo (range)	Yes	mTESE	NR	▪ IG: 157/307 (51.1%) ▪ CG (hypogonadal): 25/41 (61%) ▪ CG (eugonadal): 217/388 (56%) ▪ *p* = 0.31	IG: ▪ CPR = 79/157 (50%) ▪ LBR = 60/157 (38%) CG (eugonadal): ▪ CPR = 14/25 (56%) ▪ LBR = 12/25 (48%) *p* > 0.05 (pregnancy data NR by type of treatment) CG (eugonadal): ▪ CPR = 105/217 (48%) ▪ LBR:94/217 (43%)	NR
Hussein et al. (2013) [40]	RC	Turkey	*N* = 612 Mixed population of NG eugonadal and NG hypogonadal men with idiopathic NOA (mean FSH levels: 6.4 mIU/mL; TT levels < 300 ng/dL in 140 patients) ▪ IG: *n* = 496 ▪ CG: *n* = 116	26.7 (4.9)	CC 50 mg EOD for 2 wk, followed by various regimens based on initial response to CC and follow-up FSH and TT levels, including: ▪ Group 1: CC only (varying doses ranging from 50 mg Q3D to 75 mg EOD) for 6.4 ± 2.4 mo (*n* = 372), if follow-up FSH levels increased > 50% from baseline and TT target (500–800 ng/dL) achieved ▪ Group 2: CC 50 mg EOD + 5000 IU uhCG 2 × /wk for 4.1 ± 2.4 mo (hCG dose adjusted to achieve TT target) (*n* = 62), if suboptimal increase/no increase in follow-up LH and TT levels ▪ Group 3: Stop CC, and 5000 IU uhCG 1 × /wk + 75 IU hMG 1 × /wk added for 4.2 ± 1.1 mo (hCG dose adjusted for TT target; *n* = 46), if no obvious increase in FSH, LH and TT levels despite CC adjustments ▪ Group 4: Stop CC, and uhCG 5000 IU 1 × /wk + hMG 75 IU 1 × /wk added for 4.2 ± 1.1 mo (hCG dose adjusted for TT target; *n* = 16), if TT paradoxically decreased after increased CC dosage	5.4 mo (range: 3–9)	Yes	mTESE	IG: ▪ Group 1: 41/372 (11%) ▪ Group 2: 7/62 (11.3%) ▪ Group 3: 4/46 (8.7%) ▪ Group 4: 2/16 (12.6%) ▪ CG: 0/116 (0%)	IG: ▪ Group1: 191/331 (57.7%) ▪ Group 2: 31/55 (56.4%) ▪ Group 3: 22/42 (52.4%) ▪ Group 4: 8/14 (57.1%) ▪ CG: 39/116 (33.6%) *p* < 0.05 (CG vs IG)	NR	None
Shiraishi et al. (2016) [41]	CS	Japan	*N* = 21 HG eugonadal (mean FSH: 22.1 IU/L; mean TT: 453 ng/dL) with idiopathic NOA and failed mTESE	32.2 (3.1)	uhCG 5000 IU 3 × /wk for 1 mo, followed by uhCG 5000 IU 3 × /wk + rFSH 150 IU 3 × /wk for 3 mo	4 mo	No	Repeat mTESE	NR	2/21 (9.5%)	1/21 (4.8%)	Acne: 3/21 (14.3%)
Hu et al. (2018) [42]	RC	China	*N* = 35 HG eugonadal (FSH > 5.5 IU/L; TT > 9.4 nmol/L) with idiopathic NOA and failed TESE, all with HYPO ▪ IG: *n* = 25 ▪ CG: *n* = 10	▪ IG: 25.8 (3.4) ▪ CG: 26.6 (3.3)	Goserelin 3.6 mg single dose for 1 mo, followed by goserelin 3.6 single dose + u 2000 IU 1 × /wk for 1 mo, and lastly, goserelin 3.6 mg 1 × /mo + uhCG 2000 IU 1 × /wk + hMG 150 IU 2 × /w for 4 mo	6 mo	Yes	cTESE	NR	▪ IG: 2/25 (8%) ▪ CG: 0/10 (0%)	NR	Goserelin alone: erectile dysfunction, libido loss, asthenia) 10/25 (40%), resolved with hCG
Amer et al. (2019) [43]	RC	Egypt	*N* = 1395 ▪ HG (FSH > 8 mIU/mL) ▪ IG: *n* = 426 ▪ CG: *n* = 969	▪ + SR: 37.2 (9.7) ▪ − SR: 36.4 (7.6)	Various regimens (doses not specified), including hCG (type not specified) + hMG (*n* = 131; anti-estrogen (*n* = 66); testosterone (*n* = 79); HP-uFSH or rFSH (*n* = 22); hCG + hMG + anti-estrogen (*n* = 41); hCG + hMG + testosterone (*n* = 34); anti-estrogen + testosterone (*n* = 37) + AI (*n* = 6); hCG + hMG + FSH + anti-estrogen + T (*n* = 10)	NR	Yes	mTESE	NR	▪ IG: 118/426 (27.7%) ▪ CG: 332/969 (34.3%) ▪ *p* = 0.21 Stratified by hormonal therapy regimen: ▪ hCG + hMG: 33/131 (25.2%) ▪ Anti-estrogen: 16/66 (24.2%) ▪ T: 21/79 (26.6%) ▪ FSH: 9/22 (40.9%) ▪ hCG + hMG + anti-estrogen: 10/41 (24.4%) ▪ hCG + hMG + T: 6/34 (17.6%) ▪ Anti-estrogen + T: 15/37 (40.5%) ▪ AI: 2/6 (33.3%) ▪ hCG + hMG + FSH + anti-estrogen + T: 6/10 (60%) ▪ *p* = 0.198	NR	NR
Amer et al. (2020) [44]	PC	Egypt	*N* = 40 ▪ HG (mean FSH: 25.4 IU/L) with failed mTESE ▪ IG: *n* = 20 ▪ CG: *n* = 20	▪ IG: 35.9 (5.4) ▪ CG: 36.2 (4.3)	T-enanthate 250 mg 1/wk for 1 mo, followed by T-enanthate 250 mg 1/wk + hCG 5000 IU (type not specified) 1/wk + HP-uFSH 150 IU 3 × /wk for 3 mo	4 mo	Yes	mTESE	NR	▪ IG: 2/20 (10%) ▪ CG: 0/20 (0%) *▪ p* = 0.07	NR	NR
Guo et al. (2020) [45]	RC	China	*N* = 184 ▪ HG (mean FSH levels: 29.1) with non-mosaic KS ▪ IG: *n* = 134 ▪ CG: *n* = 50	30.2 (4.8)	2000 IU hCG every 2 d	3 mo	Yes	mTESE	NR	▪ IG: 58/134 (43.3%) ▪ CG: 22/50 (44%)	CPR: ▪ IG: 22/49 (44.9%) ▪ CG: 9/20 (45%) *▪ p* = 0.99 LBR: ▪ IG: 15/49 (30.6%) ▪ CG: 7/20 (35%) *▪ p* = 0.69	NR
Peng et al. (2022) [46]	RC	China	*N* = 569 HG (mean FSH levels: 18.1 IU/L) ▪ IG: *n* = 395 ▪ CG: *n* = 174	▪ IG: 30.0 (28–33) ▪ CG: 32.0 (28.0–35.0)	hCG (type not specified) 2000 IU Q2D for 1 mo, followed by adjustments based on follow-up FSH levels: ▪ If FSH > 11.1 IU/L, treatment continued with 2000 IU hCG Q2D for 1 mo ▪ If FSH 0.7–11.1 IU/L), hCG 2000 IU + HP-uFSH Q2D for 2 mo	2–3 mo	Yes	mTESE	▪ IG: 27/395 (6.8%) ▪ CG: 0/174 (0%)	▪ IG: 115/368 (31.2%) ▪ CG: 34/174 (19.5%) *▪ p* = 0.006	▪ LBR: IG = 54/107 (50.5%) ▪ CG = 14/31 (45.2%) *▪ p* = 0.752	NR
Laursen et al. (2019) [47]	CR	Denmark	*N* = 1 NG eugonadal (FSH 11.6 IU/L; TT 18.2 nmol/L); cryptorchidism	28	rhCG 1560 IU 2 × /wk for 1 mo, followed by (i) dose adjustment as needed to keep TT between 23–29 nmol/L or (ii) rFSH added (150–225 IU) 2 × /wk if follow-up FSH levels < 1.5 IU/L	40 wk	No	No	Few ejaculate sperm (5 cell-sleepers stored with motile sperm)	NA	▪ Singleton delivery from ICSI using ejaculated sperm	NR
Esteves et al. (2024) [48]	RC	Brazil	*N* = 616 hypogonadal (TT < 350 ng/dL), näive of previous SR; NG (FSH ≤ 12 IU/L) *n* = 295; HG (FSH > 12 IU/L) *n* = 321 ▪ IG: *n* = 291 CG: *n* = 325	▪ IG: 35.0 (33–39) CG: 32.0 (32.0–39.0)	rhCG 2080 IU 2 × /wk for 1 mo, followed by (i) dose adjustment as needed to keep TT > 350–900 ng/dL and (ii) rFSH added (150 IU) 3 × /wk if follow-up FSH levels < 1.5 IU/L and (iii) AI (anastrozole) added (1 mg/d) if follow-up TT/E2 ratio < 10	4.7 months (IQR: 3.0–8.0)	Yes	mTESE	18 (2.7%) out of 670 patients scheduled for mTESE (13 IG and 9 CG); these patients were excluded as mTESE was canceled, and histopathology data for confirming NOA was unavailable	▪ IG: 182/291 (62.5%) ▪ CG: 167/325 (51.4%) *p* = 0.005	NR	10.3% (30/291), most related to injection site reactions (15 patients)

*AI* aromatase inhibitor, *CC* clomiphene citrate, *CG* control group, *CPR* clinical pregnancy rate, *CR* case report, *CS* case series, *cTESE* conventional testicular sperm extraction, *d* day[s], *E2* estradiol, *EOD* every other day, *FSH* follicle-stimulating hormone, *hCG* human chorionic gonadotropin, *HG* hypergonadotropic, *HGH* hypergonadotropic hypogonadal, *hMG* human menopausal gonadotropin, *HP* highly purified, *HYPO* hypospermatogenesis, *ICSI* intracytoplasmic sperm injection, *IG* intervention group, *KS* Klinefelter syndrome, *LBR* live birth rate, *LH* luteinizing hormone, *MA* maturation arrest, *mo* month[s], *mTESE* microdissection testicular sperm extraction, *NA* not applicable, *NG* normogonadotropic, *NOA* non-obstructive azoospermia, *NR* not reported, *OPR* ongoing pregnancy rate, *PC* prospective cohort study, *PR* pregnancy rate, *Q#D* every # days, *RC* retrospective cohort study, *rFSH* recombinant follicle-stimulating hormone, *rhCG* recombinant human chorionic gonadotropin, *SD* standard deviation, *SR* sperm retrieval, + *SR* positive sperm retrieval, − *SR* negative sperm retrieval, *T* testosterone, *T/E2 ratio* testosterone to estradiol ratio, *TESA* testicular sperm aspiration, *TESE* testicular sperm extraction, *TT* total testosterone, *Tx* treatment, *uFSH* urinary follicle-stimulating hormone, *uhCG* urinary human chorionic gonadotropin, *wk* week[s]

**Fig. 3:**
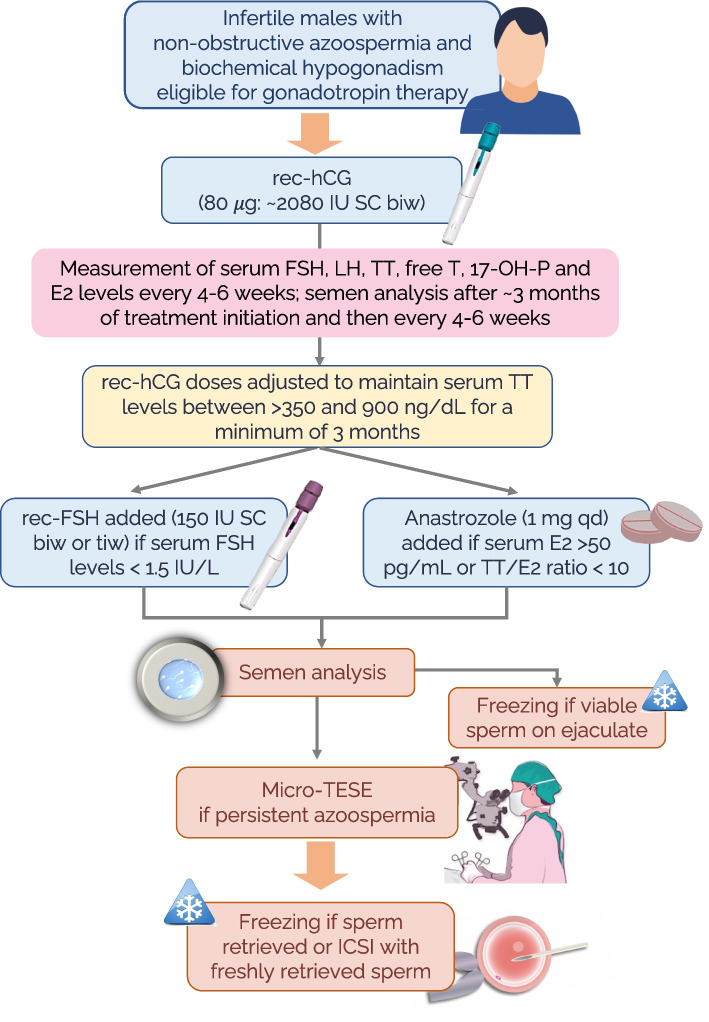
The Esteves gonadotropin treatment protocol for infertile males with non-obstructive azoospermia and hypogonadism. The treatment involves the off-label use of human chorionic gonadotropin (hCG) alone or in combination with follicle-stimulating hormone (FSH). Given the off-label nature of the treatment, patients must provide signed informed consent before initiating therapy. Subcutaneous injections of choriogonadotropin alfa (recombinant human chorionic gonadotropin [rhCG], 250 μg/0.5 mL prefilled pen for injection) in doses of 80 μg (~ 2080 IU), are self-administered twice weekly. The dose is adjusted to keep the total testosterone (TT) level > 350 and up to 900 ng/dL. If the serum FSH level falls below 1.5 IU/L during rhCG stimulation, patients are also given recombinant FSH (follitropin alfa [rFSH], 300 IU/0.5 mL, using a prefilled multidose pen ready for injection). The rFSH is administered at a dose of 150–225 IU two (biw) to three (tiw) times a week, concurrent to the rhCG therapy, for at least 3 months. An aromatase inhibitor is prescribed off-label if the estradiol (E2) levels exceed 50 pg/mL or if the TT (ng/dL) to E2 (pg/mL) ratio (T/E2 ratio) falls below 10. The aromatase inhibitor is given orally (e.g., anastrozole, 1 mg daily) to keep the estradiol levels below 50 pg/mL and the T/E2 ratio above 10. Patients are monitored with hormone measurements (serum FSH, luteinizing hormone [LH], E2, TT, free testosterone, and 17-hydroxy-progesterone [17-OH-P]) and liver enzyme measurements for those taking aromatase inhibitors every 3–4 weeks. Semen analysis is carried out 3 months after starting the treatment and then every 4 weeks for patients who continue therapy for > 3 months. If viable sperm are found in any semen analysis during treatment, sperm cryopreservation is carried out. If not, patients undergo microdissection testicular sperm extraction (micro-TESE) after ≥ 3 months of treatment. ICSI, intracytoplasmic sperm injection; qd, once daily. Adapted with permission from Elsevier from Esteves SC, Achermann APP, Miyaoka R, Verza S Jr, Fregonesi A, Riccetto CLZ. Clinical factors impacting microdissection testicular sperm extraction success in hypogonadal men with nonobstructive azoospermia. Fertil Steril. 2024 Jun 22:S0015-0282(24)00544–2. https://doi.org/10.1016/j.fertnstert.2024.06.013.

Our treatment protocol – the Esteves protocol [1, 16, 21, 47, 48] – primarily involves the off-label use of rhCG to increase ITT production (Fig. 3). Our experience indicates that hCG treatment increases circulating testosterone levels for most patients [16, 17, 21, 47, 48]. This increase in testosterone levels has the beneficial effect of resetting the elevated baseline FSH levels to normal levels. This FSH reset may improve Sertoli cell function by increasing the expression of FSH receptors [23]. Importantly, it is worth noting that some patients experience a significant decrease in FSH levels during hCG treatment due to the negative feedback of testosterone on the pituitary [16, 17, 23, 48]. In such cases, rFSH is added to the hCG regimen when FSH levels fall below 1.5 IU/L [16, 17, 21, 48]. Patients undergo monthly hormonal assessments during treatment, and if the testosterone-to-estradiol ratio falls below 10, an aromatase inhibitor is added to the treatment regimen [17, 21, 48].

#### Sperm DNA fragmentation

Sperm DNA fragmentation (SDF) refers to breaks in the DNA strands that compromise sperm chromatin integrity, potentially impairing normal embryo development and the health of offspring [49, 50]. SDF is commonly observed in infertile men and can originate in the testis due to a faulty apoptotic mechanism or arise from oxidative stress during sperm transit through the male genital tract [51, 52]. Treatment with exogenous FSH appears to reduce sperm apoptosis and improve the quality of the acrosome, axoneme, and chromatin [51, 53, 54]. This effect is likely mediated through the stimulation of DNA synthesis in spermatogonia and preleptotene spermatocytes, as well as its action on Sertoli cells, which support the survival of premeiotic germ cells [6]. The effectiveness of FSH therapy is more notable in individuals with the homozygous N polymorphism of the FSH receptor (FSHR p.N680S) compared to those with the S allele (p.N680S). This suggests that the SNP FSHB − 211G > T genotype demonstrates the highest responsiveness to therapy [55]. This observation aligns with the established interaction between FSH and its receptor, which mediates the effect of FSH on Sertoli cells. The FSHR is susceptible to single nucleotide polymorphisms (SNPs) that can influence receptor sensitivity and, consequently, the therapeutic response [56].

While hormone therapy with exogenous FSH administration has been shown to reduce SDF in men with idiopathic infertility, the effects of combining FSH and LH-activity regimens remain uncertain. However, considering the distinct molecular actions of LH and hCG on Leydig cells, where hCG primarily induces cAMP and increases intracellular Ca2 + , leading to upregulation of genes encoding steroidogenic enzymes [1], while LH activates proliferative and survival signals through ERK1/2 or AKT phosphorylation), it is reasonable to hypothesize that such combinations could also influence SDF. This hypothesis, however, requires further investigation.

### Future research directions

Advancements in pharmacological therapy hold promise for mitigating infertility in men. However, several critical areas related to the use of gonadotropins in male infertility treatments require further investigation. Real-world data studies and prospective clinical trials are essential to evaluate the efficacy and safety of gonadotropins in hypogonadal infertile males with idiopathic oligozoospermia or NOA. Within these categories, there is a need to identify which patients may benefit from gonadotropin treatment and determine the optimal treatment regimens and durations. Another area of interest is establishing serum testosterone thresholds that support optimal spermatogenesis, emphasizing the need for a novel classification of infertile males to stratify patients based on endocrine and semen analysis parameters. Recently, the APHRODITE criteria, a new classification system for infertile men with testicular dysfunction, was introduced [57]. The system aims to enhance patient stratification and optimize hormonal therapy, potentially improving fertility outcomes and advancing the field's understanding of male infertility. . Lastly, given the potential impact of sperm/seminal microbiome and sperm DNA fragmentation on semen quality and reproductive outcomes [6, 49–55, 58–60], research is needed to investigate the effects of LH-containing gonadotropins on these parameters. These efforts are anticipated to improve patient care and promote the discovery of innovative pharmacological treatment options for male infertility.

## Conclusions

Studies suggest that gonadotropins with LH activity have a generallypositive therapeutic effect on alleviating male infertility, particularly in patients with HH and NOA. Leydig cells in the testes express LHCGRs, which both LH and hCG can bind. HCG formulations are preferred for increasing ITT production in hypogonadal men, including those with HH, idiopathic oligozoospermic, and NOA, due to their lower costs and broader availability compared with rhLH. Therapy with hCG alone or combined with hMG, urinary FSH, or rFSH has been shown to restore spermatogenesis to varying degrees in HH patients, often enabling natural conception or assisted reproduction. In NOA patients, who typically exhibit low intrinsic testicular function and decreased ITT levels, hCG treatment holds promise to improve sperm retrieval outcomes. Boosting testosterone levels by hCG helps suppress the elevated FSH levels commonly seen in NOA patients and mitigates Sertoli cell receptor desensitization caused by chronic FSH elevation. Treatment with LH-activity gonadotropins may enable oligozoospermic men to achieve biological fatherhood through intrauterine insemination or natural conception, instead of requiring in vitro fertilization. For NOA patients, it may allow ICSI treatment. However, the efficacy of LH-activity gonadotropins in treating male infertility needs further validation through large-scale, well-designed studies. Further research should focus on identifying the most suitable candidates for treatment, optimizing gonadotropin treatment protocols,and clarifying the distinct roles of LH and hCG in Leydig cell function. Additionally, exploring the clinical utility of rhLH in male infertility remains an important area of investigation.

## Supplementary Information

Supplementary Material 1.

## Data Availability

The datasets supporting the conclusions of this article are included within the article and its additional files.

## Abbreviations

ART: Assisted reproductive technology
cAMP: Cyclic adenosine monophosphate
CG: Chorionic gonadotropin
FSH: Follicle-stimulating hormone
GnRH: Gonadotropin-releasing hormone
H: Human
HH: Hypogonadotropic hypogonadism
MG: Menopausal gonadotropin
HP: Highly purified
ICSI: Intracytoplasmic sperm injection
ITT: Intratesticular testosterone
IVF: In vitro fertilization
LH: Luteinizing hormone
NOA: Non-obstructive azoospermia
OR: Odds ratio
R: Recombinant
