# Supplementary material for: The role of luteinizing hormone activity in spermatogenesis: from physiology to clinical practice
Source: Reprod Biol Endocrinol. 2025 Jan 13;23(Suppl 1):6. doi: 10.1186/s12958-024-01333-4 (PMC11726975; doi:10.1186/s12958-024-01333-4)
Supplement: Supplementary file 1 — Supplementary Material 1. [file 12958_2024_1333_MOESM1_ESM.pdf]

ONLINE SUPPLEMENT

Esteves SC*, Humaidan P

The role of luteinizing hormone activity in spermatogenesis: From physiology to clinical implications

Supplemental Table 1. Characteristics of commercially available human chorionic gonadotropin (hCG) preparations

Proportion of total immunoreactivity (%)	**Urinary-derived hCG[a]**	**Recombinant hCG**
Intact bioactive hCG (%)	50–96	>99
Hyperglycosylated hCG (%)	0.5–4.0	<0.1
Free beta subunit (%)	2.4–8.0	<0.1
Beta-core fragment[b] (%)	1.2–58.0	—
Epidermal growth factor[c]	4–204	—

[a] Varies by brand.

[b] Degradation product of hCG.

[c] Epidermal growth factor is a contaminant (ng/5000 IU).

* Corresponding author at s.esteves@androfert.com.br
